# Intakes of Dairy and Soy Products and 10-Year Coronary Heart Disease Risk in Korean Adults

**DOI:** 10.3390/nu16172959

**Published:** 2024-09-03

**Authors:** Sinwoo Hwang, Ae Wha Ha

**Affiliations:** 1 Hamilton Glaucoma Center, Shiley Eye Institute, Viterbi Family Department of Ophthalmology, University of California San Diego, La Jolla, CA 92093, USA; sinwoohwang@health.ucsd.edu; 2 Department of Food Science and Nutrition, College of Science and Technology, Dankook University, Cheonan 31116, Republic of Korea

**Keywords:** coronary heart disease, cow’s milk, dairy, soymilk, soy products, adult

## Abstract

Dairy and soy products are healthy food. However, studies have reported conflicting results associating their intake with coronary heart disease (CHD). Thus, this study determined the association between intake of dairy or soy products and 10-year CHD risk. Participants aged 40~69 years were grouped into those who consumed dairy products (more or less than twice a week) and those who consumed soy products (more or less than twice a week). Ten-year CHD risk (%), atherogenic index (AI), and atherogenic index of plasma (AIP) were calculated. The CHD risk, according to the level of dairy and soy product intake, was expressed as an odds ratio (OR) and a confidence interval (CI). Significant differences were observed in sex, age, education, income, and living area according to dairy intake frequencies, whereas only age showed significant differences according to soy products’ intake frequencies. Relative effects of Framingham Risk Score (FRS) factors on 10-year CHD risk in Korean adults were found to be significant in the order of age, high-density lipoprotein cholesterol (HDL-C), smoking, blood total cholesterol (TC), systolic blood pressure (SBP), diabetes, and sex. Overall, participants who consumed dairy products ≥2/week had a significantly lower OR of 10-year CHD risk compared to those who consumed dairy products <2/week after adjusting for confounding factors (OR: 0.742, 95% CI: 0.619 to 0.890). Otherwise, intake of soy products ≥2/week tended to decrease the OR of 10-year CHD risk, although the decrease was not statistically significant. In conclusion, Korean adults who consumed dairy products ≥2/week had higher HDL-C and lower 10-year CHD risk than those who consumed dairy products <2/week. However, these results did not appear when consuming soy products.

## 1. Introduction

Coronary arteries are blood vessels that supply blood and nutrients to the heart. When the coronary vessel walls become narrowed and blocked, coronary heart disease (CHD), like angina pectoris and myocardial infarction, occurs [1]. According to a report from Statistics Korea, approximately 1/3 of adults aged 30 years or older have hypertension. In addition, the number of patients with dyslipidemia has doubled in the past 10 years, indicating that the incidence of CHD is continuously increasing along with the mortality rate [2]. 

Risk factors for CHD include non-correctable factors (such as genetic factors, age, and gender) and correctable factors such as diet, smoking, high blood pressure, diabetes, and hyperlipidemia. Among them, dietary factors such as excessive intake of foods high in saturated fatty acids or cholesterol have a significant impact on hypercholesterolemia and CHD [3]. 

Dairy and soy products are two food categories that have been extensively studied for their potential effects on CHD. While dairy products have long been a staple of the Western diet, soy products have gained popularity in recent years due to their potential health benefits. Both dairy and soy products contain bioactive compounds that may play a role in the development and prevention of CHD. Dairy products are healthy foods that contain several nutrients, such as proteins and calcium. However, studies have reported conflicting results regarding the association between dairy product consumption and CHD. Some studies have shown that saturated fatty acids in dairy products can increase the risk of CHD [4,5,6,7], while other studies have found that various physiologically active peptides in dairy products play a beneficial or neutral role in CHD [8,9,10,11].

A growing number of people are switching from cow’s milk to soy milk because of health concerns, milk allergies, and a preference for vegetarian diets [12]. Soy products are low in cholesterol. In addition, they contain high-quality vegetable proteins and unsaturated fatty acids. Some studies have suggested that consumption of soy products may help lower cholesterol levels and reduce the risk of CHD and other conditions such as liver disease, inflammation, and bone disease [13,14,15,16,17,18,19]. However, an association between soymilk consumption and CHD has not yet been clearly established. 

Koreans traditionally consume various bean-based products in their diets. These include tofu, fermented soy products such as doenjang (soy paste), and other bean dishes such as soft tofu. Soy milk has become increasingly popular in recent years due to its nutritional benefits and its suitability for people with lactose intolerance. However, the consumption of soy milk in Korea is not as high as that of other soy products, such as soybean paste or tofu. 

Several indices have been developed as tools for predicting CHD. The atherogenic index (AI) and atherogenic index of plasma (AIP) are indicators for predicting CHD by changes in serum levels of the lipid profile [20,21]. Framingham Risk Score (FRS) has been developed from the Framingham Cohort Study [22]. The FRS is a 10-year CHD risk prediction tool that incorporates factors such as gender, age, smoking status, diabetes status, blood pressure, total cholesterol (TC), and high-density lipoprotein cholesterol (HDL-C) [23]. So far, FRS is the most widely used risk assessment indicator because it is a useful tool for identifying and managing patients at high risk of CHD among healthy individuals [22,23,24,25,26,27,28]. 

Therefore, the relationship between dairy or soy product consumption and CHD risk was then determined by comparing various CHD risk indices with data from the 2012–2016 Korea National Health and Nutrition Examination Survey (KNHANES).

## 2. Materials and Methods

### 2.1. Data Collection

The food frequency questionnaire (FFQ) used in KNHANES is a method to investigate the frequency of servings of 63 food items for a certain period. It estimates the daily consumption pattern of the participant based on the average intake frequency and amount. The semi-quantitative FFQ used in KNHANES calculates food and nutrient intake frequency and average intake per serving [26]. This study was conducted in accordance with the ethical principles of the Declaration of Helsinki after it was approved by our Research Ethics Review Committee (KNU_IRB_2020-64, Kongju National University, Korea). 

### 2.2. Participants 

Of a total of 39,156 participants of 2012–2016 KNHANES, participants under the age of 19 (n = 12,089) and those who were diagnosed with CHDs (stroke (n = 582), myocardial infarction (n = 245), angina pectoris (n = 515)) were excluded. Of a total of 25,725 participants remaining, those under the age of 40 (n = 8316) and those missing answers for dairy products or soy products consumption frequency (n = 1067) were excluded. Of a total of 16,342 participants remaining, only participants aged 40–69 years (n = 8747), those with food intake frequency survey data (dairy and legume products), were finally selected. Participants were grouped based on their frequency of dairy products or soy products consumption as follows: dairy products group (those who consumed dairy products more than twice a week (n = 3247) or less than twice a week (n = 5612)) and soy products group (those who consumed soy products more than twice a week (n = 665) or less than twice a week (n = 8194)). 

### 2.3. Dairy or Soy Products Intake Frequency

Among FFQ data, types of dairy products included cow’s milk, yogurt (liquid), and yogurt (solid). For soybean products, soymilk, tofu (solid bean curd), bean curd (liquid), and soybean (cooked) were included. Those food items were divided into 9 frequencies as follows: rarely, once a month, 2~3 times a month, once a week, 2~4 times a week, 5~6 times a week, once a day, twice a day, and 3 times a day. Each intake frequency of solid yogurt, liquid yogurt, and cow’s milk was converted into intake frequency per week and summed up as the weekly frequency intake of dairy products. For soy products, the intake frequency of tofu (solid type), soft tofu (liquid type), braised soybean, and soy milk was converted into intake frequency per week and summed up as the weekly frequency intake of soy products. Finally, intakes of dairy or soy products were divided into two groups: less than twice a week and more than twice a week (as frequent intake). Given the frequency of intake, the amount of intake was calculated using the following formula: daily intake (g/day) = frequency (times/day) × reference amount [1 serving] × ratio of the reference amount. 

### 2.4. Dairy or Soy Products Intake and General Characteristics

Of general characteristics, age (40~49 years, 50~64 years), sex, drinking habit (less or more than once a month), education level (middle school or lower, high school, university or higher), average monthly house income (low, middle–low, middle–high, high), and living area (large city, middle and small city, rural area) were compared among groups. Body mass index (BMI, kg/m^2^) was used to define underweight (<18.5), normal weight (18.5–24.9), and obese (≥25.0).

### 2.5. Dairy or Soy Products Intake and CHD Risk (FRS, AI, and AIP)

The FRS is a tool for estimating the 10-year CHD risk. Its score is based on risk factors of age, sex, SBP, TC level, HDL-C level, smoking status, and diabetes status [20]. Age was divided into 5-year units from 40 years old. The score for each age group (by sex) was reflected differently depending on sex (scores ranging from 0 to 8). Blood HDL-C level was divided into 5 groups (scores ranging from −3 to 5). Blood TC was also divided into 5 groups (scores ranging from −2 to 3). The presence or absence of smoking status was scored as 0 or 2. SBP of each of the five levels was scored, ranging from −1 to 7, considering whether hypertension was treated or not. The presence or absence of diabetes was scored 0 or 4. The 10-year CHD risk (%) was calculated by the FRS total point, which was the sum of the above six indicators (ranging from <1 to 30). Both AI and the AIP were measures of blood lipid profiles as indices of CHD risk. AI was calculated by TC divided by HDL-C. AIP was calculated by log transformation after plasma triglyceride (TG) divided by HDL-C level [27,28]. 

### 2.6. Statistics

Data analysis and cleaning procedures were conducted using SAS 9.4 (SAS Institute, Cary, NC, USA), which considered stratified multistage probability sampling design and the integrated weight of KNHANES. Continuous variables are expressed as mean and standard deviation using proc survey multiple regression. Categorical variables are presented as percentages reflecting frequency and weight by proc survey frequency analysis. Statistical significance was determined by the χ^2^-test. The correlation between dairy products or soy products intake and variables was analyzed using proc survey multiple regression. A post hoc test was performed using the Bonferroni test, which considered the design effect of complex KNHANES sampling. To calculate the risk ratio of variables for weekly dairy products or soy products intake frequency, proc survey logistic regression was used after adjusting for age, sex, energy intake, and obesity. The results are expressed as OR and 95% CI.

## 3. Results

### 3.1. Participants’ Intakes of Dairy and Soy Products

The average frequency of dairy products was 3.73 times/week, and the average daily intake was 83.9 g/d (Table 1). Cow milk consumption and intake frequency were the highest. In the group that consumed dairy products <2/week, the total intake of dairy products was 10.6 g/d, and the frequency was 0.5 times/week. In the group that consumed dairy products ≥2/week, frequency and intake of dairy products were 6.6 times/week and 150 g/d, respectively.

For soybean products, the intake was 37.8 g/d, and the frequency was 2.1 times/week. The intake amount according to the type of soybean product was similar. The frequency was in the order of tofu, boiled soybean, soft tofu, and soy milk. In the group that consumed soybean products less than twice a week, the frequency and amount of soybean intake were 0.8 times/week and 14.9 g/d, respectively. In the group that consumed soybean products more than twice a week, the frequency and amount of soybean intake were 4.8 times/week and 85.6 g/d, respectively.

### 3.2. General Characteristics of Participants

Men accounted for 49.3% of total participants (Table 2). More women consumed dairy products more than twice a week than men (*p* < 0.001). High education, income, and living in a large city were associated with high intakes of dairy products. Among the variables studied, only house income showed a significant difference among those with different intake frequencies of soy products. 

### 3.3. Relative Effects of FRS Factors on 10-Year CHD Risk

Ten-year CHD risk was determined by six risk factors: age, TC, HDL-C, SBP, diabetes, and smoking (Table 3). Ten-year CHD risk increased with age (*p* < 0.0001), TC (*p* < 0.0001), and SBP (*p* < 0.0001) but decreased with higher HDL-C (*p* < 0.0001), non-smokers (*p* < 0.0001), and non-diabetics (*p* < 0.0001). The relative magnitude of the effect of each CHD risk factor calculated using a standardized regression coefficient (beta) was found to be significant in the order of age, HDL-C, smoking, TC, SBP, diabetes, and sex (sex was not included in the six factors). In addition, there was no autocorrelation in that the determination coefficient (R^2^), indicating the explanatory power of this model was 73.5%, and Durbin–Watson was 2.155. The multiple collinearity test showed that all tolerance values were above 0.1 and VIF values were below 10, confirming that the regression model had no multiple collinearity problem.

### 3.4. Ten-Year CHD Risk and Relative Factors According to Dairy or Soy Products’ Intake

Since the FRS calculation formula already included age, data were analyzed after controlling for sex, education, house income, and living area (Table 4). Among 10-year CHD risk factors, HDL-C (*p* < 0.05) and diabetes status (*p* < 0.05) showed significant differences according to dairy products’ intake frequencies. Compared to those with dairy product intake < 2/week, those with dairy product intake ≥ 2/week had significantly higher HDL-C levels (51.9 mg/dL) and a lower rate of smoking. There were no significant differences in TC, HDL-C, SBP, or diabetes according to dairy products’ intake frequencies. The 10-year CHD risk estimated FRS score (*p* < 0.05) was significantly lower in those with dairy products intake ≥ 2/week than in those with intake < 2/week. Those with dairy product intake ≥ 2 /week had significantly lower rates of high CHD risk (2.7%) than those with dairy product intake < 2/week. No significant difference was observed in CHD risk levels among those with different intake frequencies of soy products. AIP, another indicator of CHD risk, was also significantly lower in those with dairy product intake ≥ 2/week than in those with dairy product intake < 2/week. Among 10-year CHD risk factors, there were no significant differences in TC, HDL-C, SBP, diabetes, or smoking. However, age showed a significant (*p* < 0.05) difference according to soy product intake frequencies. AI and AIP levels showed no significant differences according to soy product intake frequencies. 

### 3.5. Ten-Year CHD Risk According to Dairy or Soy Products’ Intake Frequencies (Age and Sex)

Ten-year CHD risk level was divided into low, intermediate, and high levels according to the FRS score for sex and age (Table 5). Men showed no significant difference in CHD risk levels according to dairy or soy product intakes in all age groups. The proportion of those with a high CHD risk was significantly increased to 8.3% or 21.7% in the age group of 50–59 years or 60–69 years, respectively. Overall, 60.3%, 33.1%, and 6.6% of men were at low risk, intermediate risk, and high risk of CHD, respectively. In women of age 50, those with dairy product intake ≥ 2/week had a higher proportion of those with a low CHD risk compared to those with dairy products < 2/week. However, there were no significant differences in CHD risk levels according to dairy products’ intake frequencies in women in their 40s or 60s. No significant differences were observed in CHD risk levels according to soy product intake for any age group. Overall, 88.5%, 10.7%, and 0.9% of women were at low risk, intermediate risk, and high risk of CHD, respectively. 

### 3.6. The Odds Ratio of 10-Year CHD Risk According to Dairy or Soy Products’ Intake Frequencies

Logistic regression was performed to analyze the effects of sociodemographic characteristics and dairy or soy products’ intake frequencies on 10-year CHD risk (Table 6). The OR and 95% CI were indicated. Model 1 had no correction. Model 2 was corrected for age, sex, smoking, house income, living area, obesity, and daily energy intake. When independent variables overlap with correction variables, they are excluded from correction variables. According to the age group, OR of 10-year CHD risk was significantly higher in the age group of 50–69 years compared to 40–49 years before or after correcting variables (model 1 OR 5.485 vs. model 2 OR 9.869). In terms of sex, the OR of 10-year CHD risk for men was significantly higher than that for women before or after correction (model 1 OR 8.899 vs. model 2 OR 21.019). Smokers had 1.974 times higher OR of 10-year CHD risk than non-smokers before correction (model 1) and 6.811 times after correction (model 2). Compared to those with college graduation, the ORs of CHD risk were higher in those with lower education levels (elementary school graduation OR 2.14 vs. middle school graduation OR 1.569 in model 2). Compared to the high-income group, the OR of 10-year CHD risk in the low-income group was higher. The OR of 10-year CHD risk had no significant difference according to the living area after correction. The obesity group had 2.3 and 2.6 times significantly higher ORs of 10-year CHD risk compared to the normal group both before and after correction, respectively. Overall, the group that consumed dairy products more than three times a week had a significantly lower OR of 10-year CHD risk compared to those who consumed non-dairy products both before and after correction (model 1: OR 0.671, 95% CI 0.567~0.793; model 2: OR 0.742, 95% CI 0.619~0.890). The group that consumed soy products more than three times a week tended to decrease the OR of 10-year CHD risk, although the decrease was not statistically significant. 

### 3.7. Correlation between 10-Year CHD Risk and Nutrient Intakes or CHD Indices (AI and AIP) 

After adjusting for sex, age, income, education, living area, and daily energy intake, both men (*p* < 0.001) and women (*p* < 0.001), higher consumption of dairy products was significantly associated with lower 10-year CHD risk (Table 7). In women, there was a correlation between 10-year CHD risk and calcium (*p* < 0.05, negative direction) and CHO (*p* <0.05, positive direction) intake, but not men. There was a positive correlation between 10-year CHD risk and other CHD indicators, such as AI (*p* < 0.001) and AIP (*p* < 0.001) in both men and women. 

## 4. Discussion

This study analyzed 10-year CHD risk based on FRS by dividing consumption of dairy products and soy products into two or more times a week and less than two times a week in comparison with other CHD indices. In terms of sociodemographic characteristics, there were significant differences in sex, age, education, income, and living area according to dairy products’ consumption frequencies (Table 1). There were no significant differences according to sex, education, income, or living area for soy products’ consumption frequencies, although the proportion of those consuming soy products ≥ 2/week was higher in participants over age 50. These results are consistent with a previous study showing that dairy product consumption is higher in women, younger age groups, and those with higher socioeconomic levels [29]. Our results are also consistent with a previous study showing that there is no correlation between soy product consumption and sex but age [30]. The relationship between consumption frequencies of dairy products or soy products and 10-year CHD risk was determined after adjusting for sex, age, education, income, living area, and energy intake (in the case of soy products intake, only age and energy intake were used as correction variables) (Table 2 and Table 3). 

The 10-year CHD risk was calculated as a total score based on the FRS [20]. In Korea, it has been reported that the estimated 10-year CHD risk for both men and women is higher when TC is applied instead of low-density lipoprotein cholesterol (LDL-C) in the FRS equation [23,24,25]. Therefore, the 10-year CHD risk (%) was calculated using TC. This study validated the determination coefficient (R^2^) indicating FRS estimation regression, the *t*-test of indices’ regression coefficients, and the fitness of the FRS model calculated by multicollinearity investigation (Table 2). In addition, FRS showed significant positive correlations with other types of CHD indicators, such as AI and AIP (Table 6). 

The 10-year CHD risk is divided into three stages: low, FRS < 10%; intermediate, FRS = 10–19%; and high, FRS ≥ 20% [21]. The average 10-year CHD risk of participants in this study was 6.6%. It was found that 74.6%, 21.8%, and 3.6% of participants had low, intermediate, and high CHD risk levels, respectively (Table 4). The results of this study showed that CHD risk was significantly lower in those who consumed dairy products more than twice a week than in those who consumed dairy products less than twice a week (*p* < 0.05). A similar pattern was also observed for another CHD risk indicator, AIP (*p* < 0.05). Among FRS-related factors, only HDL-C showed a significant difference according to dairy products’ consumption (*p* < 0.05). These results explain that higher HDL-C can induce a lower CHD risk in those who consume dairy products more than twice a week. 

Although many studies have shown that the consumption of dairy products and soy products can decrease the risk of metabolic syndrome [31,32,33,34], the correlation between the consumption of dairy products or soy products and 10-year CHD risk has not yet been clearly established. Low HDL-C increases the CHD risk, whereas higher HDL-C has a preventive effect on CHD [8,9,10,11]. A study on Korean postmenopausal women has shown that participants who consume appropriate amounts of dairy products have higher HDL-C levels [35]. Mechanisms known to be associated with preventive effects of dairy products and dairy products consumption on CHD are mainly as follows: (1) dairy products and their consumption can reverse the LDL-C transport pathway, increase HDL-C levels, and further inhibit the production of inflammation [36,37]; (2) butyric acid, a saturated fatty acid unique to dairy products, has a low tendency to be stored in fat tissue [38]; (3) dairy products proteins contain many bioactive peptides (BAPs) that can inhibit cholesterol absorption and increase HDL-C levels [39]. 

We examined whether there was a difference in CHD risk levels (low, intermediate, high) according to dairy product and soy product consumption by sex and age (Table 4). Only in women aged 50–59, the proportion of those in the “low and intermediate” level was significantly higher in those with dairy products consumption two or more times a week. In addition, it was observed that only women with a lower intake of dairy products, calcium, and proteins had a higher 10-year CHD risk after adjusting for age, education, living area, income, and energy intake (Table 6). These results suggest that consumption of dairy products two or more times a week might be beneficial for reducing CHD risk, especially in women in their 50s. 

Previous studies have shown that there are differences in food selection according to sex, with women being more sensitive to food selection for health as they get older. The proportion of those who practice a healthy diet is also higher in women than in men [40]. Unlike men, women aged over 40 with diabetes have higher carbohydrate intake but lower protein intake [41,42]. With advancing age, women show decreased consumption of dairy products and increased metabolic syndrome [32]. Another study has shown that women with low HDL-C have higher intakes of plant-based carbohydrates and proteins and lower intakes of animal-based proteins [42]. Considering those results, appropriate dairy product consumption in postmenopausal women may help not only with protein status but also improve their HDL-C to prevent CHD. 

Previous studies have suggested that excessive consumption of dairy products and dairy products can lead to obesity and increased risk of CHD [4,5,6,7]. However, as observed in this study, 34% of adults in Korea consumed milk two or more times a week (average 100 g/d), of which only 13% consumed the recommended one cup (200 mL) of milk per day. Therefore, because this study was conducted on Koreans whose average daily milk intake is low, it is expected that there will be differences from the results of Western studies where milk intake is high [41]. 

Logistic regression analysis showed that there was no statistically significant difference in the OR of 10-year CHD risk according to soy products’ consumption frequencies before (model 1) or after (model 2), adjusting for confounding factors such as sex, age, smoking, education, income, living area, and obesity (Table 6). 

Research studies on soy product consumption and CHD are limited, and study results are also conflicting. Some studies claimed that soy products contain isoflavones and polyphenols that can decrease blood pressure, blood sugar, and inflammation [14,15,43,44], but other studies reported no or little relationships between consumption of soy products or soy proteins and blood lipid profile [16,17,18,19]. In this study, there was no difference in the 10-year CHD risk between the group that consumed soy milk less than twice a week and the group that consumed it more than twice a week, and there was no significant difference in CHD risk between the two groups even after stratification analysis by gender and age. Some meta-analysis studies have indicated a significant inverse association between soy/soy product intake and CVD mortality, the risk of CVD, stroke, and CHD risk in some Asian populations [45,46]. Thus, soy consumption tended to be beneficial in the adjusted models, and it is possible that statistical significance could be achieved with a larger sample size. 

The importance of soy products is highlighted in that they are calcium substitutes for vegans and those with milk allergies or lactose intolerance. They are also low-cost sources of protein and energy for populations with insufficient milk supply [12,13]. 

Soy products are sources of plant-based proteins with insufficient essential amino acids such as methionine compared to dairy products, but they are rich in dietary fiber and antioxidants [43]. In fact, one study reported that when partially replacing carbohydrates with proteins (½ soy protein + ½ milk protein) in dietary compositions, blood pressure and CHD risk are reduced in prehypertension or stage 1 hypertension [44]. Further randomized clinical trials or prospective cohort studies are necessary for Koreans to elucidate the role of dairy or soy food intake in CHD risk.

The limitations of this study are as follows. First, it was a cross-sectional study that dietary intake relies on memory at the time the survey was conducted. Thus, the causal relationship between CHD risk and dietary intake of dairy or soy products cannot be established. Another limitation is that the number of participants who consumed soy products was small, but the disease and smoking rates in that group were higher than those consuming dairy products. Although variables that could affect the study results were adjusted as much as possible, there is a possibility that bias still existed. Nevertheless, the significance of this study lies in the fact that it investigated the relationship between the intake of various soy and dairy products and CHD risk. 

## 5. Conclusions

In conclusion, compared to those who consumed dairy products less than twice a week, those who consumed dairy products more than twice a week had significantly higher HDL-C and lower 10-year CHD risk before or after adjusting for confounding variables, especially in women aged 50–59. Therefore, consumption of dairy products is beneficial for reducing the risk of CHD, especially in postmenopausal women. Meanwhile, consumption of soy products was found to have no significant correlation with CHD risk. This is a cross-sectional study that provides an initial understanding of the relationship between dairy or soy consumption and CHD risk at a given time. Thus, the results should be interpreted with caution.

## Figures and Tables

**Table 1:** The intakes of dairy and soy products in participants.

**Dairy Products**						
	Total (n = 8747)		<2/week (n = 4034)		≥2/week (n = 4713)	
	Frequency (times/week)	Intake (g/day)	Frequency (times/week)	Intake (g/day)	Frequency (times/week)	Intake (g/day)
Cow’s milk	2.00 ± 0.04	60.68 ± 1.25	0.24 ± 0.01	6.96 ± 0.2	3.60 ± 0.06	109.22 ± 1.96
Yogurt (Liquid)	0.99 ± 0.03	11.80 ± 0.35	0.17 ± 0.01	1.89 ± 0.07	1.73 ± 0.05	20.76 ± 0.6
Yogurt (Solid)	0.73 ± 0.02	11.37 ± 0.39	0.12 ± 0.00	1.70 ± 0.06	1.29 ± 0.04	20.12 ± 0.68
Total	3.73 ± 0.06	83.86 ± 1.49	0.52 ± 0.01	10.55 ± 0.23	6.62 ± 0.07	150.1 ± 2.09
**Soy Products**						
	Total (n = 8747)		<2/week (n = 5092)		≥2/week (n = 2845)	
	Frequency (times/week)	Intake (g/day)	Frequency (times/week)	Intake (g/day)	Frequency (times/week)	Intake (g/day)
Soymilk	0.42 ± 0.02	11.98 ± 0.45	0.09 ± 0	2.51 ± 0.1	1.12 ± 0.04	31.79 ± 1.21
Bean curd (Solid)	0.76 ± 0.01	12.76 ± 0.25	0.35 ± 0.01	5.75 ± 0.09	1.62 ± 0.03	27.43 ± 0.6
Bean curd (Liquid)	0.39 ± 0.01	11.87 ± 0.26	0.21 ± 0	6.39 ± 0.13	0.77 ± 0.02	23.32 ± 0.67
Soybean (Cooked)	0.49 ± 0.02	1.17 ± 0.05	0.11 ± 0	0.26 ± 0.01	1.3 ± 0.05	3.06 ± 0.13
Total	2.07 ± 0.03	37.79 ± 0.69	0.76 ± 0.01	14.92 ± 0.2	4.81 ± 0.07	85.61 ± 1.45

**Table 2:** General characteristics of participants (%).

Variables	Total (n = 8747)	Dairy Products		*p*-Value ^2^	Soy Products		*p*-Value
		<2/week	≥2/week		<2/week	≥2/week	
**Sex**							
Men	49.3 ^1^	56.2	43.1	<0.0001	48.9	50.1	0.3516
Women	50.7	43.8	56.9		51.1	49.9	
**Age group**							
40~49 year	46.4	45.9	46.8	0.1023	46.7	45.8	0.3711
50~59 year	41.2	40.8	41.6		40.7	42.3	
60~69 year	12.4	13.2	11.6		12.6	11.9	
**Education** **(graduate)**							
Elementary	13.4	15.0	10.5	<0.0001	13.4	14.0	0.8789
Middle school	13.1	14.3	10.8		13.0	13.6	
High school	39.8	39.2	40.8		39.8	40.2	
≥College	33.7	31.4	37.8		33.9	32.2	
**Family income**							
low	9.3	11.2	7.5	<0.0001	9.9	8.0	0.0051
Middle–low	23.3	24.9	21.8		23.8	22.1	
Middle–high	30.6	31.2	30.0		30.7	30.2	
high	36.9	32.6	40.8		35.6	39.6	
**Living area**							
Large city	45.5	44.5	47.1	0.0136	45.6	44.0	0.6961
Middle city	37.2	37.1	37.4		37.2	37.4	
Rural area	17.3	18.4	15.5		17.2	18.6	
**Drinking**							
≥1/month	85.4	85.0	86.1	0.1828	85.5	84.1	0.3919
Obesity ^3^							
Underweight	2.2	2.2	2.3	0.2301	2.1	3.5	0.0628
Normal	62.0	61.3	63.2		61.9	63.0	
Obese	35.8	36.5	34.5		36.0	33.4	

All analyses accounted for the complex sampling design and sampling weight of the national survey. ^1^ Weighted %. ^2^ *p*-value (χ^2^ value) by χ^2^-test. ^3^ Obesity, divided by body mass index (BMI, kg/m^2^): underweight, BMI > 18.5; normal weight, 18.5 ≤ BMI ≤ 24.9; overweight and obese, BMI ≥ 25.0 (Source: Korean Society for the Study of Obesity, Obesity Treatment Guidelines 2022 and WHO, 2000, The Asia–Pacific perspective: redefining obesity and its treatment).

**Table 3:** Linear regression analysis of FRS factors and 10-year CHD risk in total participants.

Parameter	Estimate	Standardized Estimate	Standard Error	t Value	*p*-Value	Multicollinearity	
						Tolerance	VIF
Intercept	−12.254	−7 × 10^15^	0.625	−19.58	<0.0001		
Age (year)	0.255	0.331	0.007	34.99	<0.0001	0.89099	1.12235
sex (Men)	2.026	0.193	0.108	18.68	<0.0001		
sex (Women)	Ref.						
Total cholesterol (mg/dL)	0.042	0.288	0.001	26.57	<0.0001	0.91661	1.09097
HDL-cholesterol (mg/dL)	−0.131	−0.318	0.004	−31.39	<0.0001	0.90332	1.10703
Systolic blood pressure (mmHg)	0.089	0.268	0.004	21.68	<0.0001	0.91173	1.09681
Diabetes (No)	4.518	−0.223	0.302	−14.93	<0.0001	0.96743	1.03367
Diabetes (Yes)	Ref.						
Smoking (No)	−3.708	−0.294	0.181	−20.42	<0.0001	0.92948	1.07588
Smoking (Yes)	Ref.						

All analyses accounted for the complex sampling design and appropriate sampling weights of the national survey. Note: R^2^ = 0.100, adjusted R^2^ = 0.076, F = 6.505, *p* = 0.002, Durbin–Watson = 2.155. Abbreviations: B, unstandardized coefficient; SE, standard error; β, standardized coefficient; t, t-statistic; VIF, variation inflation factor.

**Table 4:** Ten-year CHD risk and relative factors according to dairy or soy product intake.

Variables	Dairy Products		Soy Products		Total
	<2/week	≥2/week	<2/week	≥2/week	
age (year) ^1^	51 ± 0.1 ^2 NS 3^	50.3 ± 0.2	50.6 ± 0.1 *	51.5 ± 0.3	50.5 ± 0.1
Total cholesterol (mg/dL)	195.4 ± 0.6 ^NS^	196.7 ± 0.7	195.9 ± 0.5 ^NS^	195.7 ± 1.9	195 ± 0.5
HDL-cholesterol (mg/dL)	49.7 ± 0.2 *	51.9 ± 0.3	50.3 ± 0.2 ^NS^	50.7 ± 0.6	50 ± 0.2
Systolic blood pressure (mmHg)	118.8 ± 0.3 ^NS^	117.7 ± 0.3	118.4 ± 0.2 ^NS^	118 ± 0.7	118 ± 0.2
Diabetes status (Yes %)	8.3 *	6.0	7.1 ^NS^	7.0	7.1
Smoking status (Yes %)	24.6 ^NS^	24.1	22.2 ^NS^	22.6	22.5
FRS score	4.4 ± 0.1 *	3.8 ± 0.1	4.0 ± 0.1 ^NS^	4.1 ± 0.2	4.1 ± 0.1
10-year CHD risk (%)	7.3 ± 0.1 *	6.2 ± 0.1 *	6.6 ± 0.1 ^NS^	6.5 ± 0.1	6.7 ± 0.1
Low % ^4^	72.4 ***	78.2	74.6	73.6	73.6
Intermediate %	23.3	19.0	21.8	21.2	21.2
High %	4.3	2.7	3.6	5.2	5.2
AI	3.15 ± 0.02 ^NS^	3.03 ± 0.02	3.11 ± 0.02 ^NS^	3.07 ± 0.06	3.07 ± 0.02
AIP	0.42 ± 0.01 *	0.37 ± 0.01	0.40 ± 0.01 ^NS^	0.39 ± 0.02	0.39 ± 0.01

All analyses accounted for the complex sampling design and appropriate sampling weights of the national survey. ^1^ FRS components. ^2^ Mean ± SE for continuous variables and % for categorical variables. ^3^ *p*-value was derived from proc survey multiple regression (*t*-test) for continuous variables, after adjusting for sex, education, house income, living area, and chi-square test for categorical variables: NS: not significant. *, *p* < 0.05; ***, *p* < 0.001. ^4.^ 10-year CHD risk level: low, FRS < 10%; intermediate, FRS = 10–19%; high, FRS ≥ 20%. AI (atherogenic index) = (blood non-HDL cholesterol)/blood HDL cholesterol. AIP (atherogenic index of plasma) = log (blood TG/blood HDL cholesterol). FRS: Framingham Risk Score; CHD: coronary heart disease.

**Table 5:** Ten-year CHD risk level according to dairy and soy products intake by age and sex.

Sex/Age	Classification	Dairy Products		Soy Products		Total
		<2/week	≥2/week	<2/week	≥2/week	
Men						
40~49	Low	76.9 ^1NS2^	78.6	77.3 ^NS^	79.6	77.5
	Intermediate	20.9	20.6	21.0	18.7	20.8
	High	2.1	0.8	1.7	1.7	1.7
50~59	Low	49.1 ^NS^	50.1	48.9 ^NS^	53.8	49.4
	Intermediate	41.5	44.1	42.8	36.9	42.3
	High	9.4	5.9	8.2	9.3	8.3
60~69	Low	25.9 ^NS^	26.4	26.2 ^NS^	24.3	26.0
	Intermediate	52.7	51.1	52.7	47.4	52.2
	High	21.4	22.5	21.0	28.2	21.7
Total	Low	59.5 *	62.0	60.3 ^NS^	60.1	60.3
	Intermediate	33.2	33.0	33.3	31.1	33.1
	High	7.3	5.0	6.4	8.8	6.6
Women						
40~49	Low	99.7 ^NS^	99.6	99.6 ^NS^	100	99.6
	Intermediate	0.3	0.4	0.4	0.0	0.4
	High					
50~59	Low	82.4 **	88.5	84.6 ^NS^	83.2	84.4
	Intermediate	16.8	11.1	14.7	15.2	14.7
	High	0.8	0.4	0.8	1.6	0.8
60~69	Low	63.7 ^NS^	63.7	63.3 ^NS^	68.6	63.7
	Intermediate	33.3	31.0	32.9	27.2	32.5
	High	3.0	5.3	3.8	4.2	3.9
Total	Low	87.2 **	90.3	88.5 ^NS^	88.0	88.5
	Intermediate	12.0	8.7	10.7	10.7	10.7
	High	0.8	1.0	0.8	1.3	0.9

All analyses accounted for the complex sampling design and appropriate sampling weights of the national survey. ^1^ Percentage (%). ^2^ *p*-value by chi-square test. NS: not significant. *, *p* < 0.05; **, *p* < 0.01. FRS: Framingham Risk Score; CHD: coronary heart disease, CHD risk level: low, FRS < 10%; intermediate, FRS 10–19%; high, FRS ≥ 20%.

**Table 6:** The odds ratio for 10-year CHD risk among the study population.

		Model 1 ^1^		Model 2 ^2^	
		OR ^3^	95% CI ^4^	OR	95% CI
Age group	50~64	5.485	(4.620, 6.511) ^5^***	9.869	(7.360, 13.235) ***
	40~49	1	Reference	1	Reference
Sex	Men	8.899	(7.831, 10.948) ***	21.019	(15.019, 28.691) ***
	Women	1	Reference	1	Reference
Smoking status	Yes	1.974	(1.185, 3.288) ***	6.811	(4.644, 9.988) ***
	No	1	Reference	1	Reference
Education level	Elementary	3.921	(2.521, 4.924) ***	2.140	(1.444, 3.174) **
	Middle school	1.999	(2.521, 2.529) ***	1.596	(1.142, 2.417) *
	High school	0.914	(0.763, 1.094)	1.169	(0.908, 1.506) ^NS^
	University	1	Reference	1	Reference
Income	Low	1.917	(1.489, 2.598) ***	1.661	(1.142, 2.417) *
	Medium-low	1.540	(1.250, 1.896) ***	1.307	(0.965, 1.770) ^NS^
	High-low	1.011	(0.827, 1.235)	0.943	(0.727, 1.224) ^NS^
	High	1	Reference	1	Reference
Living area	Rural area	1.353	(1.097, 1.669) *	0.954	(0.737, 1.236) ^NS.^
	Middle and Small City	0.925	(0.778, 1.099)	0.855	(0.674, 1.086) ^NS^
	Large city	1	Reference	1	Reference
Obesity level	Underweight	0.381	(0.222, 0.655) **	0.340	(0.164, 0.763) **
	Obesity	2.300	(1.913, 2.765) ***	2.594	(2.045, 3.290) ***
	Normal	1	Reference	1	Reference
Dairy products intake	Yes	0.671	(0.567, 0.793) ***	0.742	(0.619, 0.890) **
	No	1	Reference	1	Reference
Soy products intake	Yes	1.298	(0.839, 2.008) ^NS^	0.732	(0.383, 1.414) ^NS^
	No	1	Reference	1	Reference

All analyses were conducted considering the complex sampling design and the sampling weights. Dairy products intake: consumed dairy products 2 times a week/or not. Soy product intake: consumed soy milk 2 times a week/or not. ^1^ Model 1: *p*-value by logistic regression (influence variable uncorrected).^2^ Model 2: *p*-value by logistic regression after adjusting for age, sex, education level, house income, living area, obesity, and smoking status (when the independent variable is a control variable, it is excluded from the control variable).^3^ OR: odds ratio. ^4^ CI: confidence interval. ^5^: * *p* < 0.05; **, *p* < 0.01; ***, *p* < 0.001. NS: not significant.

**Table 7:** Partial correlations between 10-year CHD risk and diet or other CHD indices.

	Men	Women	Total
^1^ Dairy products intake (g/d)	^2^ −0.04813 ^3^**	−0.0463 **	−0.1321 *
	0.005	0.0008	<0.001
Calcium intake (mg/d)	−0.0120 ^NS^	−0.0359 *	−0.0395 **
	0.7047	0.0106	0.0024
Fat intake (g/d)	0.02364 ^NS^	−0.01727 ^NS^	−0.0292 **
	0.1646	0.2062	0.0066
Protein intake (g/d)	0.00941 ^NS^	−0.0149	0.0434 ***
	0.58	0.2845	<0.001
Carbohydrate intake(g/d)	−0.02706 ^NS^	0.02573 *	0.064 ***
	0.1116	0.0500	<0.001
^4^ AI	0.58693 ***	0.53287 ***	0.55167 ***
	<0.0001	<0.0001	<0.0001
^5^ AIP	0.48411 ***	0.49919 ***	0.531 ***
	<0.0001	<0.0001	<0.0001

^1^ Determined using the FFQ data. ^2^ Pearson’s correlation coefficient (r) adjusted for age, income, and energy intake. ^3^ *, *p* < 0.05; **, *p* < 0.01; ***, *p* < 0.001, NS: not significant: determined by Pearson’s correlation coefficient. ^4^ AI: atherogenic index. ^5^ AIP: atherogenic index of plasma.

## Data Availability

The original contributions presented in the study are included in the article, further inquiries can be directed to the corresponding author.
